# Optical power profiles and aberrations of a non-diffractive wavefront-shaping extended depth of focus intraocular lens

**DOI:** 10.1007/s00417-024-06469-y

**Published:** 2024-04-10

**Authors:** Nuria Garzón, José Antonio Gómez-Pedrero, César Albarrán-Diego, Sara Fernández-Núñez, Sara Villanueva Gómez-Chacón, María García-Montero

**Affiliations:** 1 Dpto. de Optometría y Visión, Universidad Complutense de Madrid, Av. Arcos del Jalón 118, 28037 Madrid, Spain; 2 Dpto. de Óptica, Universidad Complutense de Madrid, Av. Arcos del Jalón 118, 28037 Madrid, Spain; 3 Dpto. de Óptica y Optometría y Ciencias de La Visión, Universitat de València, Doctor Moliner 50, Burjassot, 46100 Valencia, Spain

**Keywords:** IOL, EDoF, Power profile, Aberrometric profile, Optical quality

## Abstract

**Purpose:**

This study is to evaluate the optical characteristics of a non-diffractive wavefront-shaping intraocular lens which incorporates surface refractive modifications for shaping the wavefront in order to achieve extended depth of focus (EDoF) and to assess whether the nominal power of this IOL influences the attainable add power.

**Methods:**

A commercially available optical bench NIMO TR1504 device (LAMBDA-X, Nivelles, Belgium) was employed to obtain full optical characterization of three non-diffractive EDoF intraocular lenses with + 10 D, + 20 D, and + 30 D powers. After NIMO measurements, data were computed using a custom-made MATLAB program (Mathworks, Inc., Natick, MA, USA) to evaluate the optical quality functions, such as the point spread function (PSF), wavefront profiles, and modulation transfer function (MTF) for two pupil sizes: 3 mm and 4.0 mm.

**Results:**

The non-diffractive EDoF intraocular lens showed a central serrated power profile behavior with additions of + 2.00 to + 2.50 D over the nominal power. Higher order aberrations were found to be driven mainly by the spherical aberration, with almost null comatic influence. Optical quality metrics showed good values, better for a 3 mm pupil compared to a 4.5 mm one, as expected. The three IOL powers tested showed a very similar behavior in terms of power and aberrometric profiles, with minimal to null differences related to the nominal power.

**Conclusion:**

The non-diffractive wavefront-shaping EDoF intraocular lens achieves a near addition up to + 2.50 D aiming for an extended range of vision, almost independently of the base power.


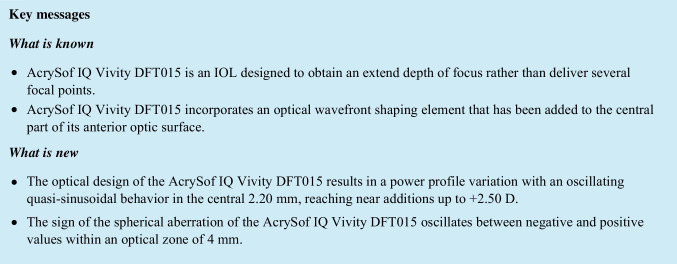


## Introduction

Several optical designs of intraocular lenses (IOL) are currently available, aiming to provide the best solution for patients who wish to achieve independence from spectacles following cataract surgery. Monofocal IOLs provide clear vision, usually for far distant. Therefore, the use of spectacles is necessary for other distances. On the other hand, multifocal IOL models can provide clear vision at several distances, depending on their optical configuration. Nevertheless, they can also generate visual disturbances, such as halos and glare. Frequently, these issues become a primary source of discomfort or dissatisfaction for patients following uncomplicated cataract surgery [1].

The latest designs applied to intraocular lenses are the extended depth of focus (EDoF) lenses. Compared to conventional lenses, EDoF lenses produce an elongated caustic along the optical axis, which is a pattern of elongated focused light rather than a simple focal point. This characteristic contributes to increasing the lens’ depth of focus [2]. EDoF IOL technology has the potential to bridge the gap between monofocal IOLs and multifocal IOLs. These lenses aim to enhance visual acuity at intermediate distances, potentially leading to fewer or less severe visual disturbances and improved contrast sensitivity [2, 3].

The extended depth of focus can be achieved through various technologies, including diffractive optics (e.g., Symfony by Johnson & Johnson, CA, USA), a small aperture design working a pinhole (e.g., IC-8 by AcuFocus, Inc.), a bioanalogic design that emulates the crystalline lens without optics (e.g., WIOL-CF by Medicem), or by introducing spherical aberration (e.g., LuxSmart by Bausch & Lomb GmbH, Germany).

An alternative EDoF design is the AcrySof IQ Vivity DFT015 lens manufactured by Alcon (Alcon, Fort Worth, TX, USA), which employs refractive surfaces for shaping the wavefront and achieving the EDoF. The AcrySof IQ Vivity DFT015 IOL employs an innovative wavefront shaping technology (X-WAVE), which includes two smoothly transitioning surface elements consisting of a circular toroidal-shaped central modification in the anterior surface of the lens responsible for stretching and shifting the wavefront. The first surface transition element is a slightly elevated, smooth plateau (approximately 1 μm) that introduces a delay to a segment of the wavefront as it passes through the IOL. This delay contrasts with the more advanced wavefront passing through the IOL outside of the central surface transition elements [4].

The main goal of this study was to evaluate the radial profiles, spherical aberration (SA) values and high order aberrations (HOA) associated with the AcrySof IQ Vivity DFT015 lens. Furthermore, we aimed to assess whether the nominal power of this IOL influences the attainable add power, considering + 10.00, + 20.00, and + 30.00 D lenses. Addressing these two objectives is essential in determining IOL indications and in the management of patients receiving this type of IOL.

## Material and methods

This study was conducted at the laboratory facilities of the Optics and Optometry Faculty of the Universidad Complutense de Madrid, Spain.

### Intraocular lens

AcrySof IQ Vivity DFT015 is an IOL designed to obtain an extend depth of focus which provides functional vision at both far and intermediate and near distance. The Acrysoft IQ Vivity, described by the manufacturer as an extended depth of focus (EDoF) lens, is a C-loop single piece IOL with a 6 mm optic and an overall diameter of 13 mm made in hydrophobic acrylic material with a refractive index of 1.55 at 35 °C and a low Abbe number of 37. The manufacturer provides a limited information regarding spherical aberration, claiming that is also designed with negative spherical aberration to compensate for the positive spherical aberration of the cornea (-0.2 µm) [5], without providing the necessary data of the optical zone related to the spherical aberration value [6]. The lens has an aspheric anterior surface with a central modification consisting in the addition of an optical element with a toroidal profile and is presented by the manufacturer as a non-diffractive wavefront shaping element (X-WAVE™ technology). In the lens design, two anterior surface transition elements are used: a surface transition element, a slightly elevated plateau (~ 1 µm) that stretches the wavefront resulting in a continuous extended focal range and surface transition element, and a small curvature change that shifts the wavefront so that all the energy is usable. Without this shift, half of the extended focal range would be placed in front of the retina (myopic result), and half behind (hyperopic result), with a loss of effective focal range. For that reason, the small curvature change moves the focalized range shifting the light from the hyperopic direction to the myopic direction so that the entire light energy is effective. The two surface transition elements forming the central toroidal-shaped modification work synergistically and simultaneously to create a continuous extended focal range [7]. According to the patent, this central circular toroid responsible for the focus extension has an inner edge 0.55 mm away from the optical axis of the lens, whereas the outer edge is located 1.11 mm away from the optical axis, so it occupies the central 2.22 mm of the central IOL optic [8, 9].

### Power profile mapping and wavefront analysis

The NIMO TR1504 device (LAMBDA-X, Nivelles, Belgium) employed in this study measures the refractive effective power and complete wavefront aberrations of monofocal, toric, and multifocal IOLs. This device operates on the phase-shifting Schlieren principle [10]. By integrating this principle with the phase-shifting method commonly utilized interferometry [11], the NIMO system can assess distortions in light beams and utilize this information to compute the optical lens’ power characteristics. It also conducts wavefront analysis, encompassing up to the 13th order of the Zernike coefficients. The measurement light source exhibits a radiance peak at 546 nm, which closely aligns with the spectral relative luminance efficacy peak of the human visual system, positioned at 555 nm in photopic conditions.

Once the fringe pattern has been captured, the NIMO TR1504 calculates the data, allowing for a detailed measurement of power distribution within any selected optic zone. The instrument’s software also facilitates wavefront analysis through Zernike polynomial decomposition at the desired optical diameter of the lens. In this study, the optical zone diameter was set to 4.5 mm to investigate variations in power and spherical aberration across a broad range of pupil diameters, reflecting different usage scenarios and physiological characteristics of users. To compute the addition, we calculated the difference between the radial power measurements obtained and the nominal power provided by the lens manufacturer.

The main parameters measured in this study were the radial power profiles expressed in diopters, the root mean square (RMS) of total high order aberrations (HOAs) expressed in microns (from the third to thirteenth order), and the Zernike coefficient values related to the spherical aberration [from Z(4:0) to Z(8:0)] expressed in microns, for different optical diameters. The RMS was studied for 3 mm and 4.5 mm.

The measurements were carried out following the protocol published by Gomez-Pedrero et al. [12] for IOLs.

To evaluate the optical quality, the point spread function (PSF) and the modulation transfer function (MTF) were computed from the wavefront data measured by the NIMO device. The computation was performed using a custom-made MATLAB program (Mathworks, Inc., Natick, MA, USA), within a cornea ISO2 eye model, following the principles of Fourier Optics to simulate the optical quality parameters (wavefront, PSF, MTF). For this simulation, all Zernikes up to the order 5th as well as SA of the order 6th and 8th were included.

## Results

**Fig. 1:**
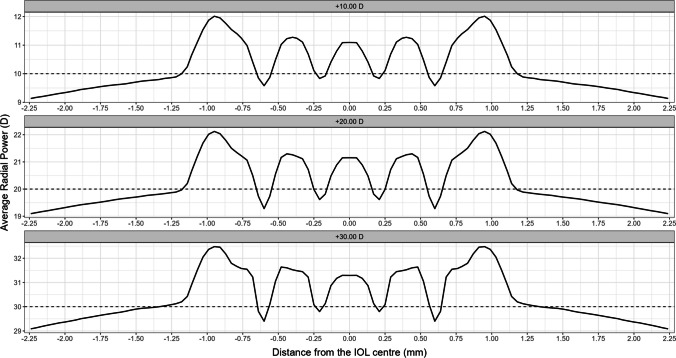
AcrySof IQ Vivity DFT015 power profiles for the three measured powers (+ 10.00 D (up), + 20.00 D (middle), + 30.00 D (bottom)), representing the average power in diopters (vertical axis) measured at different distances in millimeters from the IOL center (horizontal axis) up to a 4.50 mm optical zone. The dashed line for each plot represents the nominal power

Figure 1 shows the average power profiles obtained from 10 consecutive measurements for each of the three IOL powers.

In Fig. 1, we observe that the optical power varies across the lens. In the central 2.20 mm zone of the lens (± 1.10 mm from the center), the power profiles show a pattern of increasing and decreasing values resembling a sine wave. Specifically, for the + 10.00 D and + 20.00 D IOLs, there is an increase of approximately + 2.00 D above the nominal power, while for the + 30.00 D IOL, this increase reaches + 2.50 D. Examining the power profiles from the periphery of the lens towards its center, this increase starts around 1.25 mm away from the center, peaks at 1.00 mm, and then gradually decreases, returning to the nominal value at approximately 0.60 mm from the center. Another smaller increment occurs at about 0.50 mm from the center, adding approximately + 1.40 D for the + 10.00 D and + 20.00 D IOLs and + 1.50 D for the + 30.00 D IOL, before decreasing again towards the nominal value at around 0.25 mm from the center. In the very central zone of the lens (within 0.12 mm from the center, i.e., the central 0.24 mm optic zone), there is an additional increase in power of approximately 1.25 D above the nominal power, with a slight further increase for the + 30.00 D IOL.

**Fig. 2:**
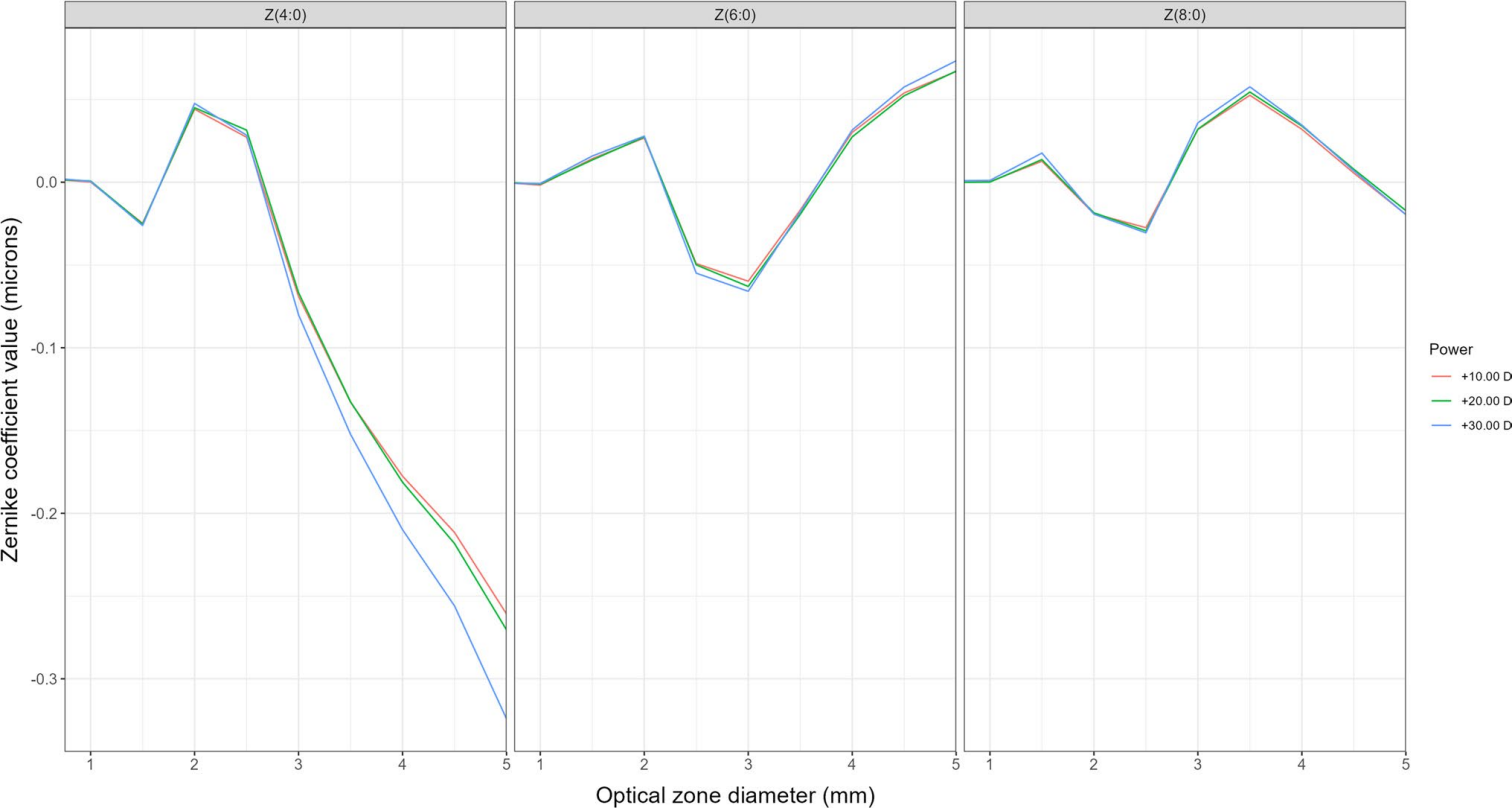
AcrySof IQ Vivity DFT015 spherical aberration profiles for the three measured powers (+ 10.00 D (red line), + 20.00 D (green line), + 30.00 D (blue line)), representing Zernike coefficient value in microns (vertical axis) measured for different IOL optical zones in millimeters (horizontal axis) up to a 5.00 mm optical zone

Figure 2 shows the spherical aberration profile from the center of the measured IOLs up to an optical zone of 5.00 mm, considering spherical aberration Zernike coefficients up to the 8th order. Higher orders of SA have not been included given its null contribution.

As can be seen from Fig. 2, the sign of the spherical aberration oscillates between negative and positive values when the diameter of the optical zone increases up to 4 mm, and then it steadily decreases driven by the coefficient of the Z(4:0) Zernike polynomial. It is almost equal for the three nominal powers measured. For optical zones higher than 4 mm, the total spherical aberration remains negative given the big contribution of the primary spherical aberration Z(4:0). The -0.20 microns of primary spherical aberration Z(4:0) claimed by the manufacturer is valid for a 4.00 /4.50 mm optic zone, as can be seen in the left plot of Fig. 2.

**Fig. 3:**
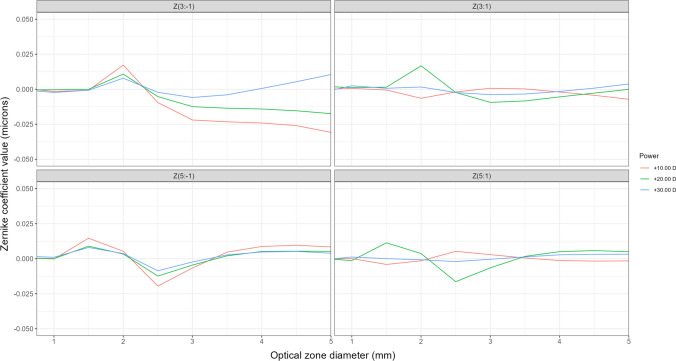
AcrySof IQ Vivity DFT015 coma aberration profiles for the three measured powers (+ 10.00 D (red line), + 20.00 D (green line), + 30.00 D (blue line)), representing Zernike coefficient value in microns (vertical axis) measured for different IOL optical zones in millimeters (horizontal axis) up to a 5.00 mm optical zone

Figure 3 shows the primary and secondary coma profile from the center of the measured IOLs up to an optical zone of 5.00 mm.

As depicted in Fig. 3, the AcrySof IQ Vivity shows low values of coma when measured in a perfect centered position (note the narrow range in vertical axis, from + 0.050 to + 0.050 microns). Both primary vertical coma Z(3:-1) and primary horizontal coma Z(3:1) present a subtle increase for an optical zone of 2 mm, coinciding with the location and size of the toroidal optical element over the anterior surface of the Vivity IOL.

**Fig. 4:**
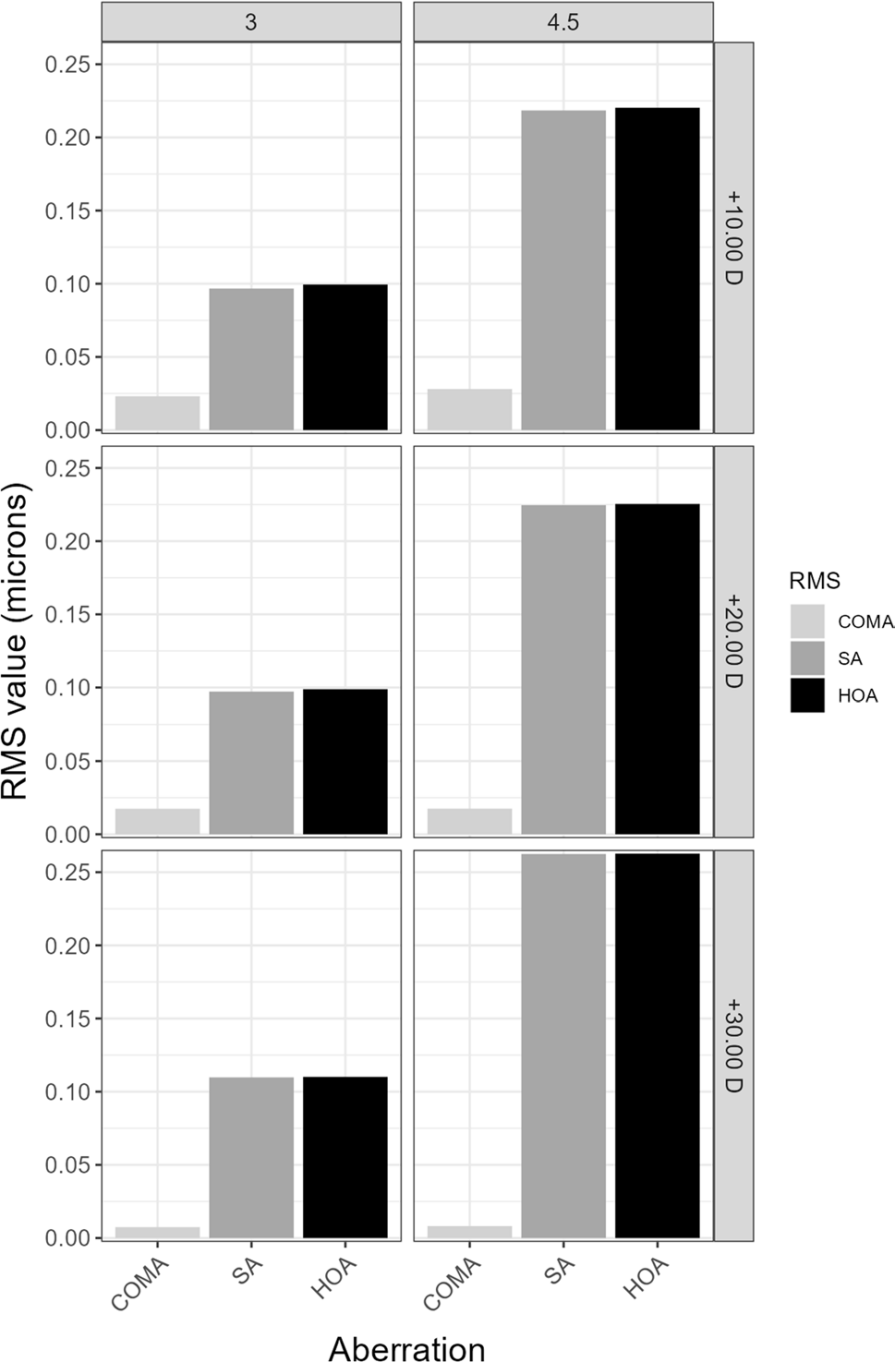
Root mean squared values (RMS) for higher order aberrations (HOAs) considering orders 3 to 8 for the three powers. SA: spherical aberration

Figure 4 shows root mean squared values for higher order aberrations (considering all terms from the 3rd order up to 8th order) and also for coma and spherical aberrations.

Figure 4 shows the almost null influence of coma aberration in the total amount of HOA when the AcrySof IQ Vivity is centered. RMS values for HOA are almost the same than RMS values for spherical aberration, which indicates that this is the predominant aberration presented by the lens.

**Fig. 5:**
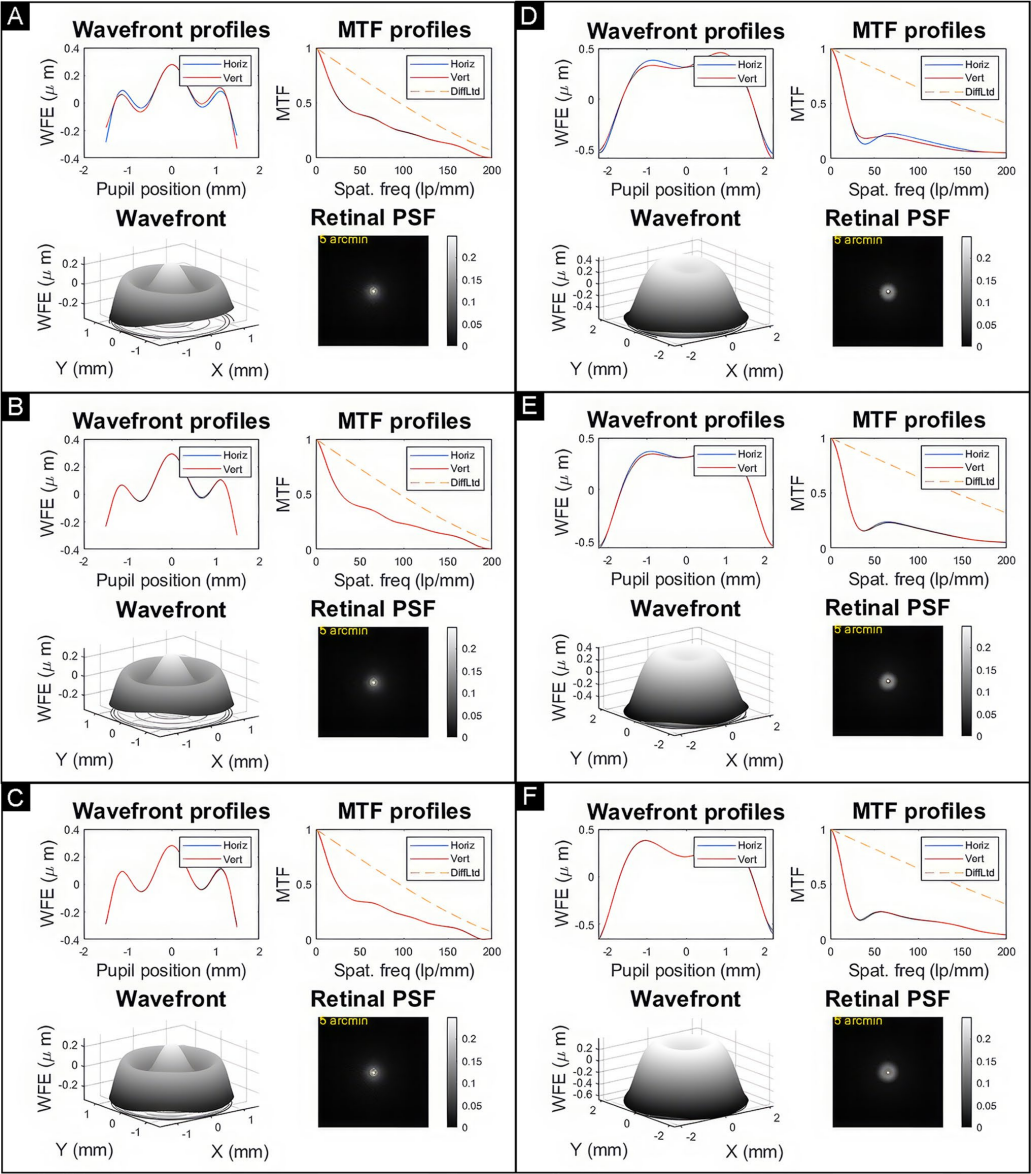
Wavefront profiles, modulation transfer function (MTF), wavefront surface, and the point spread function (retinal PSF) computed from the wavefront aberrations at 3.00 mm and 4.5 mm for the three nominal power evaluated in this study (+ 10.00 D 3 mm (**A**), + 20.00 D 3.0 mm (**B**), + 30.00 D 3.0 mm (**C**), + 10.00 D 4.5 mm (**D**), + 20.00 D 4.5 mm (**E**), + 30.00 D 4.5 mm (**F**)) considering a cornea ISO2

Figures 5 presents the simulated results of optical quality functions. The figures display wavefront profiles, modulation transfer function, wavefront, and the point spread function, all computed from wavefront aberrations at 3 mm and 4.5 mm for the three nominal powers evaluated in this study.

Figure 5 depicts MTF values around 0.4 for a spatial frequency of 50 lp/mm with a 3 mm pupil, alongside a cut-off frequency of approximately 200 lp/mm, which remains notably similar across the three tested powers. Upon considering a larger pupil of 4.5 mm, the cut-off frequency experiences an increase, while the MTF for a 50 lp/mm spatial frequency decreases to approximately 0.2. The wavefront displays an oscillating pattern for the 3 mm pupil, consistent with the power profile map illustrated in Fig. 1. Conversely, for a 4.5 mm pupil, the wavefront aberration significantly escalates towards the periphery of the lens, reaching a peak-valley value roughly twice that obtained for the 3 mm pupil. This increase in wavefront aberration leads to a notable degradation of the MTF and an enlargement of the PSF, as depicted in Fig. 5. Regarding computed retinal PSF, the pattern for a 3 mm pupil shows a high concentration of light distribution in the center, with minimal annular dispersion surrounding it. For a 4.5 mm pupil, the central peak of light appears brighter, but with a slightly higher spread of light surrounding it compared to a 3 mm pupil.

## Discussion

The advantage of extended-range lenses over other multifocal intraocular lens designs, in terms of reducing the occurrence of dysphotopsias or other disadvantages, has resulted in a fast proliferation of these types of lenses in the market. However, the American National Standards Institute (ANSI) has published clinical criteria for defining extended depth of focus (EDoF) IOLs [13]. It is worth noting that not all commercially available lenses, even if commonly referred to as EDoF, meet the ANSI criteria. However, the AcrySof IQ Vivity DFT015 model has been demonstrated to meet these criteria in clinical trials [14].

The primary feature of the lens under evaluation, in comparison to others available on the market, is an aspheric anterior surface with a central modification consisting in the addition of an optical element with a toroidal profile, resembling an axicon with a circular ring as described by McLeod [15], and is presented by the manufacturer as a non-diffractive wavefront shaping element (X-Wave technology). The optical fundamental of X-Wave technology is to engrave a toroid, with the rotation axis coaxial with the optical axis of the lens in one of the lens surfaces. The effect of this toroidal surface is to break the continuity of the wavefront. As a result of this discontinuous wavefront, the lens presents an extended depth-of-focus without the halos present when diffractive surfaces are employed.

To our knowledge, this is the first study to show the power profiles for the Vivity™ lens as well as the spherical aberration and coma across its entire surface, computed from the power maps measured by deflectometry.

The analysis of the average power profile obtained in this study (Fig. 1) reveals that the optical power in the periphery is lower and more stable than in the center, with an oscillating (or waving-like) pattern in the central zone up to 2.20 mm. The measured power profiles closely align with the manufacturer’s stated design: For all the three lenses studied, the power at the optical center is 1 D above the nominal power, and then oscillates, reaching this maximum power around 0.35 and 0.87 mm away from the optical center. Afterwards, the power peaked at 1 mm and then steadily decreased to the nominal power at, approximately, 1.25 mm away from the optical center. We hypothesize that these two zones could correspond to the beginning and end of the toroidal-shaped optical modification on the central anterior surface of the IOL. Furthermore, the power increase at the IOL center (extending 0.12 mm from the IOL center, within the central 0.24 mm optic zone) could correspond to the manufacturer’s intended change in central curvature to avoid the hyperopic part of the extended focal range, resulting in an additional power of approximately 1.25 D above the nominal power. Compared with other extended-range lens designs, such as the RayOne EMV lens [16] (Rayner Intraocular Lenses, Ltd, UK) or the ISOPure lens [17] (BVI-Physiol laboratory, Belgium), the design of the Vivity™ lens is entirely novel. This distinction arises from the fact that the aforementioned lenses show continuous power profiles that exhibit an increase in power higher than the nominal value, either in the peripheral or central zone, contingent upon whether the lens’ spherical aberration is positive or negative. This stands in contrast to the oscillating power design of the Vivity™ model. The addition achieved with the Vivity lens is between + 2.00 and + 2.50 D above the nominal value, depending on this nominal power, which could allow the patient not only to have spectacle independence in intermediate vision, but also in most daily activities, especially with electronic devices that can be used beyond 40 cm, as clinical studies have shown [4, 18]. Schmid et al. [19] in a study in which they estimated the extended range of focus for various lenses established that it was 1.7 D for 3 and 4.5 mm for Vivity™. This value is close to the addition found in our study for the lens of + 20.00 D, being the lens power evaluated by Schmid of + 22.00 D. This addition is higher than that reported for other EDoF lenses such as the Tecnis Eyhance [20] (Johnson & Johnson, CA, USA), ISOPure [17] or LuxSmart [19] (Bausch & Lomb GmbH, Germany). The 0.30 D difference in addition between our results (+ 2.00 D Add for a + 20.00 D IOL) and those by Schmidt et al. (1.70 D Add for a + 22.00 D IOL) could be due to almost two reasons. First, the different measurement methodology: We directly measure the power profile from deflectometry, whereas Schmidt et al. derive addition by the difference in peak location in the through-focus MTF for a 50 lp/mm spatial frequency. Second, Schmidt et al. used an in situ model eye according to ISO 11979, with NaCl (*n* = 1.337) which was heated to 35 °C, while our simulations were performed with a Cornea ISO2. For all these reasons, our results were not directly comparable to Schmidt’s, even though the difference is low (0.30 D).

In relation to primary spherical aberration, it has been observed that, for the three nominal powers analyzed (+ 10.00, + 20.00, and + 30.00 D), the values remain similar up to the central 3 mm. It is from this point onward that the + 30.00 D lens exhibits a slight difference, likely stemming from a peripheral design alteration to accommodate the higher power. The reported -0.20 µm value for the spherical aberration of this lens corresponds to an optical zone ranging from 4 to 4.55 mm, depending on the nominal power of the IOL. For the sixth Z(6:0) and eighth order Z(8:0) spherical aberrations, the results are consistent across all three powers. Positive values are observed in the central 2 mm for both aberrations, transitioning to negative values up to approximately the 3 mm zone before increasing back to positive values. These alterations in spherical aberration align with the regions where changes in the power profiles are observed, likely associated with the presence or absence of the optical element with a toroidal profile, to achieve the extended depth of focus. The aberrometric changes associated with the position of the central ring render the aberrometric design more complex than in other designs, where SA changes tend to be more continuous, lacking abrupt variations [17, 21, 22].

Regarding the primary [Z(3:1)] and [Z(3:-1)] and secondary [Z(5:1)] and [Z(5:-1)] coma, their influence is minimal, as the positive and negative RMS peaks align with the area where the ring is situated. In any case, these maximum values are approximately 0.025 microns or even smaller. This limited impact of these aberrations becomes evident when analyzing higher order aberrations. For both 3 and 4.5 mm, the HOAs values closely resemble the spherical aberration values for various orders, indicating that spherical aberration is the predominant factor with significant weight in the aberration analysis. Baur et al. [23] assessed higher-order aberrations in various lenses and observed that the Vivity™ lens exhibited a symmetric distribution of HOAs from the center of the lens. Furthermore, Schmid et al. [21] determined that SA was the sole significant Zernike aberration in this lens design, in agreement with our results. However, they did not investigate the changes in SA as a function of the optical zone, as conducted in the present study.

Aberrations reported in our work by means of the Zernike coefficient values for the AcrySof IQ Vivity DFT015 IOL, shown in Figs. 2–3, represent values for SA and coma at each specific distance from the lens center. Reporting single values of SA, for instance, provides limited information regarding the differences in focusing between central and peripheral light rays. For example, a value of “-0.20 microns” for an optical zone of 4 mm does not provide confident information about the behavior of light passing through the central 3 mm. This is the reason why it is so important to report aberrometric profiles and not isolated values.

Al-Amri et al. evaluated the aberrations in vivo, after implanting the lens under study [24]. The mean value of the lens implanted in their study was 21.53 ± 2.27 D, so we can compare them with those obtained with the + 20.00 D lens. The RMS HOA values obtained in vivo are slightly higher than those obtained in our study for a 3 mm pupil (0.18 vs. 0.09). This difference may stem from the real eye potentially presenting a greater number of aberrations compared to those of the ISO2 cornea, in which HOA is determined solely by spherical aberration. Furthermore, the other aberrations are not directly comparable due to differences in pupil sizes and because the aberrations are not independently separated in the clinical study.

The metrics for optical quality primarily rely on the MTF. This function determines the contrast transmitted through the model eye containing an IOL in relation to spatial frequency and pupil size. In our simulations, both the MTF value and other visual quality parameters were derived from simulations that incorporated Zernike values measured with the NIMO device and were supplemented with the values of an ISO2 cornea in the IOL plane. The initial observation in our results is that, for the three powers analyzed, the MTF values exhibit a notable similarity when comparing the same aperture. Taking the 20.00 D lenses as the reference, the Vivity™ lens yields MTF values of 0.4 at 50 lp/mm, slightly better than the values reported by Schmid et al. [25] and closely resembling those obtained by Azor et al. [26] or Baur et al. [23] for a 3 mm optical zone. For comparison purposes, a standard monofocal IOL (Tecnis ZCB00; Johnson & Johnson Surgical Vision, Inc.) shows an MTF value for 50 lp/mm around 0.5 considering a pupil of 3.0 mm [23]. As the aperture diameter increases to 4.5 mm, as expected, there is a decline in the MTF values, with results hovering around 0.2—very similar to those reported by Borkenstein et al. [27] and Schmid et al. [25]. Although our study is based on simulations using aberrometric values acquired with the NIMO TR1504, the results are entirely comparable to those obtained through other methods involving direct measurements on an optical bench. For instance, Azor’s [26] optical bench includes a model comprising an artificial cornea with a SA of + 0.27 μm for a 6 mm diameter, an iris diaphragm, and a wet cell containing saline in which the intraocular lens is immersed. Schmid [25] used an imaging test bench with a direct imaging setup using an in situ eye model with NaCl (*n* = 1337) at 35 °C to simulate a human eye was employed.

The PSF simulation computed from our NIMO results is very similar to that reported by Baur et al. [28], who claim a light pattern distribution showing minimally increased light spread compared to a monofocal IOL (the Alcon SN60WF in their study). The same result is presented by Kohnen et al. [29].

Regarding wavefront error variations, we have obtained an angular oscillating pattern of the wavefront map for a 3 mm pupil very similar to the wavefront mapping presented by Schmid and Borkenstein [21] and Baur et al. [23].

Since this is an optical bench evaluation of the AcrySof IQ Vivity DFT015 IOL, even though we can claim that this lens extends the focal range, as evidenced by the power profile presented in Fig. 1 with power increments above the nominal power around + 2.00/ + 2.50 D, our results do not provide explicit evidence about halo perception once implanted. Nonetheless, we present PSF results computed from wavefront aberrations for IOLs with + 10.00 D, + 20.00 D, and + 30.00 D for pupil sizes of 3.0 mm and 4.5 mm (see Fig. 5). The PSF for all these combinations shows a concentrated pattern of light distribution with minimal surrounding spread, suggesting a possible low impact of halo perception. This result agrees with the PSF reported for a + 20.00 D Vivity IOL by Baur et al. [28], who found a similar pattern of light distribution in the PSF of the Vivity IOL compared to a monofocal one (Acrysof SN60WF, Alcon, Fort Worth, TX, USA), both measured in an eye model with an optical bench. Regarding clinical results, the multicountry study by Bala et al. [4] found a similar incidence of perceived halos between patients implanted with the AcrySof IQ Vivity DFT015 IOL and a monofocal one (Acrysof SN60WF, Alcon, Fort Worth, TX, USA), using a quality of vision questionnaire. Additionally, the work by Kohnen et al. [29] also studied the impact of halo in patients implanted with AcrySof IQ Vivity DFT015 IOL, using a high dynamic range halo measurement system, finding similar results for the EDoF IOL and the monofocal one (Acrysof IQ, Alcon, Fort Worth, TX, USA).

Finally, regarding the possible limitations of our work, it must be considered that all the results shown in this study were obtained with the lens perfectly aligned with the optical bench axis. Therefore, further investigations are required to examine the potential impact of decentration on the optical behavior of this lens. Both the power and aberrometric profiles presented herein could change with tilting and/or decentering, potentially resulting in poorer optical outcomes. Future optical bench studies should address this possibility. In addition to optical bench studies, clinical studies involving implanted patients should analyze the impact of lens centration and tilting in visual quality.

With respect to the NIMO device used in this work to measure the optical properties of the evaluated IOL, some authors have pointed possible limitations of obtaining power maps from measuring the fringe pattern distortion using phase-shifting techniques, especially in the optical center when measuring contact lenses, and using filter options in the NIMO software. Thus, Kim et al. have reported lower repeatability measurements in the central 0.5 mm chord for bifocal and multifocal contact lenses using a specific filter configuration [30]. Nonetheless, we have previously reported very good results using the NIMO optical bench for IOL characterization, with no filter option enabled [12].

On the other hand, the main limitations associated to the used of Fourier Optics for computing the image-quality parameters are as follows: (1) The program assumes the validity of the scalar diffraction theory, (2) the PSF is computed for the far-field, and (3) the custom limitations in terms of noise and resolution of the Fast Fourier Transform (FFT) algorithm. The first assumption is valid in our case as we do not consider polarization or other vector effects. Regarding the second one, we have checked that the value of the Fresnel number is high enough to guarantee the validity of the far-field approximation [31]. Finally, we have worked with the highest possible sampling to minimize the shortcomings of the FFT algorithm.

## Conclusion

Based on the findings of this study, the incorporation of an optical element with a toroidal profile on the anterior surface, which sets the Vivity™ lens apart from other available options, plays a crucial role in shaping the lens’ power profile and aberrations. The power profile, characterized by a serrated structure, and the spherical aberration both exhibit a complex pattern with abrupt changes that precisely align with the location of this ring. Remarkably, even though the ring is just 1 micron in height, its impact on the lens’s optical characteristics is profound, and it does not result in an increase in aberrations beyond SA, providing a good optical quality. The aberrometric values obtained do not depend on the nominal value of the lens. The addition achieved in the Vivity™ lens is in the range of + 2.00 to + 2.50 D, offering a potential for a high degree of spectacle independence.
